# Optimization of Venoarterial Extracorporeal Membrane Oxygenation Weaning: Hemodynamic Targets, Predictive Indicators, and Future Algorithm

**DOI:** 10.31083/RCM43438

**Published:** 2026-01-09

**Authors:** Yanping Li, Ying Zhu, Zhuolin Wu

**Affiliations:** ^1^The Fourth School of Clinical Medicine, Zhejiang Chinese Medical University, Hangzhou First People's Hospital, 310006 Hangzhou, Zhejiang, China; ^2^Department of Critical Care, Affiliated Hangzhou First People's Hospital, School of Medicine, Westlake University, 310006 Hangzhou, Zhejiang, China

**Keywords:** extracorporeal membrane oxygenation, weaning, hemodynamics, predictive, cardiogenic shock, cardiac function

## Abstract

Venoarterial extracorporeal membrane oxygenation (VA-ECMO) is a life-saving intervention for patients with refractory cardiogenic shock or cardiac arrest. However, weaning from VA-ECMO remains challenging and significantly affects patient prognosis. This systematic review examined the multifactorial determinants underlying successful VA-ECMO weaning, highlighting the critical need for integrated evaluation of biventricular function, hemodynamic stability, and microcirculatory perfusion. Key predictive parameters encompass both macrocirculatory indices (including left and right ventricular performance) and metabolic parameters, all of which collectively inform evidence-based weaning decisions. Advanced imaging techniques and multidimensional assessment tools have emerged as promising strategies for optimizing weaning protocols. Pharmacological strategies and precise volume optimization are important for improving weaning success. However, gaps in standardized weaning protocols and bridging therapy algorithms highlight critical, unmet needs. Thus, future efforts should focus on developing dynamic predictive models that incorporate real-time hemodynamic data and on the clinical implementation of microcirculatory assessment technologies.

## 1. Introduction

Venoarterial extracorporeal membrane oxygenation (VA-ECMO) is increasingly used as temporary support for refractory cardiogenic shock. According to the Extracorporeal Life Support Organization (ELSO) international summary of statistics for 2024, despite this growth, weaning and hospital discharge rates have not shown substantial improvement. In adult patients receiving VA-ECMO for cardiac support or extracorporeal cardiopulmonary resuscitation (ECPR), the 24-hour survival rates are 57% and 38%, respectively, whereas the discharge survival rates are 47% and 31%, respectively [1]. These figures highlight persistent challenges in weaning and post-weaning care.

The definition of “successful weaning” varies across studies [2, 3, 4, 5], with some defining it as survival for 24–48 hours after weaning without the need for new assistive devices, whereas Aissaoui *et al*. [5] adopt a 30-day survival benchmark without reimplantation of mechanical support. For patients who do not survive hospital discharge after weaning, the concept of “VA-ECMO gap” has been proposed to describe this high-risk population, emphasizing the importance of identifying and addressing factors contributing to their poor outcomes [6]. The absence of standardized criteria and protocol heterogeneity further complicates clinical decision-making [7].

Therefore, when considering VA-ECMO weaning, it is very important to assess the cardiogenic causes and evaluate the function of the left and right ventricles as well as the overall cardiac function. This article provides a review of ventricular function assessment and weaning strategies for VA-ECMO, examines predictors of successful withdrawal and long-term survival, and proposes evidence-based strategies for patient selection, pre-weaning assessment, and process optimization.

## 2. Etiology-Specific Considerations for VA-ECMO Weaning

VA-ECMO improves short-term survival when conventional therapies fail, while patient outcomes under VA-ECMO are strongly influenced by etiology [8, 9]. A meta-analysis of over 29,000 cases showed mortality ranging from 35% to 76%, with the highest rates of acute myocardial infarction and cardiac arrest [9]. In contrast, five-year survival exceeds 50% in reversible conditions like myocarditis or graft failure but remains below 35% in ischemic or septic shock [8]. These findings highlight the importance of etiology-guided management: more aggressive support may be appropriate in potentially reversible conditions, whereas cautious evaluation and early consideration of alternative strategies may be warranted in cases of irreversible myocardial dysfunction.

The duration of extracorporeal membrane oxygenation (ECMO) support is another key determinant of prognosis [10, 11]. Data from the ELSO registry (n = 2699) demonstrated peak survival around day 4 of support, followed by a decline between days 4 and 12, largely attributable to complications such as infection, bleeding, and multiorgan dysfunction [11]. After day 12, survival rates plateaued, suggesting limited benefit from prolonged support in many cases. Notably, patients with reversible conditions such as myocarditis (median support duration: 154 hours; interquartile range (IQR): 96–230 hours) or post-cardiac transplantation graft dysfunction (median, 108 hours; IQR, 66–173 hours) often require extended support but may still achieve favorable outcomes [11]. These observations underscore the need to individualize weaning strategies based on the clinical trajectory, underlying disease, and early treatment response. For patients who demonstrate hemodynamic stability by day four, gradual weaning under close hemodynamic and metabolic monitoring may be appropriate.

## 3. Weaning Strategy Tailored to the Hemodynamic Phenotype of Cardiogenic Shock Under VA-ECMO Support

The key to successful weaning from VA-ECMO lies in accurately assessing the recovery of ventricular function. The cardiogenic shock phenotype, defined as congestion, may be associated with improved short-term outcomes [12]. Specifically, they can be divided into left ventricular dominant (LV-dominant), right ventricular dominant (RV-dominant), and biventricular failure types. Each phenotype exhibits significant differences in pathological mechanisms, hemodynamic manifestations, and responses to mechanical circulatory support [12]. VA-ECMO, as a form of temporary full cardiac circulatory support, can significantly alter cardiac preload and afterload, depending on cannulation strategies. Tailoring VA-ECMO weaning to the underlying ventricular phenotype (left, right, or biventricular dominant) is essential for optimal decision-making. Each phenotype entails a specific pathophysiological burden and response pattern to mechanical support.

This section systematically summarizes the hemodynamic characteristics, key assessment points, and current literature-supported weaning methods and evidence for the three phenotypes mentioned above.

### 3.1 Left Ventricular-dominant Cardiogenic Shock

The clinical features of LV-dominant cardiogenic shock include systemic hypoperfusion, elevated pulmonary capillary wedge pressure (PCWP) (usually >18 mmHg), and pulmonary congestion. The common causes include acute myocardial infarction, myocarditis, and postoperative low cardiac output syndrome [13]. In a study involving strictly selected patients with left ventricular-dominant shock, Schurtz *et al*. [14] found that even in cases with more severe baseline conditions, VA-ECMO as the initial mechanical circulatory support (MCS) technique was still superior to Impella®, showing a significant survival benefit after adjusting for confounding factors (hazard ratio = 0.25, *p* = 0.004).

The core pathophysiology of this phenotype is a significant increase in left ventricular afterload caused by retrograde aortic perfusion from VA-ECMO, which triggers a series of secondary injuries. Under pathological conditions, sustained elevation of afterload can increase ventricular wall stress, resulting in “backward failure” manifested as elevated pulmonary venous pressure, pulmonary edema, and venous system congestion. In addition, an increase in left ventricular preload can further elevate the left ventricular end-diastolic volume (LVEDV), which, through the Frank–Starling mechanism, temporarily enhances stroke volume. However, when the left ventricular contractile reserve is limited, its preload reserve is rapidly depleted, making the ventricle highly sensitive to changes in afterload [15, 16]. Notably, a simulation study found that in cases of LV-dominant failure, the relative preservation of right ventricular function may become a key risk factor for increased left ventricular load. The contraction of the right ventricle produces an “additional preload”, which further increases the mechanical burden on the failing left ventricle [17]. It was described as ventricular interdependence, wherein dysfunction of one ventricle affects the function of the other through interventricular septal mechanics, and is a critical yet often underappreciated factor during VA-ECMO weaning [17, 18]. Eventually, the pressure-volume loop (PVL) shifts to the right and expands upward, resulting in a significant increase in the pressure-volume area (PVA) and myocardial oxygen consumption [13].

Along with the increase in PVA, there is prolonged closure of the aortic valve, stagnation of blood flow in the left ventricle, and an increased risk of thrombosis. Clinically, this may present as the disappearance of the pulse pressure, pulmonary edema, and Harlequin syndrome, among other manifestations [13, 19]. On this pathological basis, implementing left ventricular unloading or venting strategies can effectively alleviate the above adverse effects. A meta-analysis including 1327 VA-ECMO patients showed that active left ventricular unloading (such as intra-aortic balloon pump (IABP) or Impella) can significantly reduce in-hospital mortality (relative risk (RR) 0.86, 95% confidence interval (CI): 0.78–0.94; number needed to treat = 17) [19]. Ezad *et al*. [13] summarized various methods of left ventricular unloading, including IABP, Impella, and atrial septostomy, and highlighted their key roles in reducing LV pressure, improving pulmonary circulatory load, and promoting myocardial recovery.

### Assessment Before Weaning in Patients With Left Ventricular Dominant Type

In LV-dominant cardiogenic shock, assessing the potential for cardiac function recovery is crucial for guiding weaning off VA-ECMO. Bedside transthoracic echocardiography (TTE) is one of the most commonly used assessment tools and is particularly important during the low-flow phase, when ECMO flow is reduced to 1–1.5 L/min. If enhanced left ventricular systolic function and improved valvular motion are observed at this stage, it suggests that myocardial function has the potential to recover [20, 21]. Ultrasound assessment should cover the structure of the left and right heart chambers, valvular function, intracardiac thrombus, pericardial effusion, and respiratory variations of the inferior vena cava (IVC), among others [20, 22, 23, 24].

Specific assessment indicators for left heart function included left ventricular ejection fraction (LVEF), left ventricular outflow tract velocity-time integral (LVOT VTI), pulse pressure (PP), PCWP, and serum biomarkers. According to the 2021 ELSO interim consensus recommendations, during low-flow states (1–1.5 L/min), a small dose of vasoactive drugs should be used to maintain a mean arterial pressure (MAP) >60 mmHg. If any of the following indicators are met, a weaning assessment trial can be attempted [25]:

• LVEF >25–35%;

• LVOT VTI >10–12 cm;

• Tissue Doppler lateral mitral annulus peak systolic velocity (TDSa) ≥6 cm/s.

Among them, LVOT VTI can serve as a continuous dynamic monitoring indicator, and its trend changes can reflect the status of left ventricular output more accurately than single measurements [23].

The pulmonary artery catheter (PAC) is a classic invasive hemodynamic monitoring tool that complements bedside ultrasound [12, 21, 26]. PAC can continuously monitor parameters such as native cardiac output, pulmonary vascular resistance, mixed venous oxygen saturation (SvO_2_), and PCWP, which help assess left heart filling status and unloading effects and guide the adjustment of assist devices (such as IABP/Impella) [27]. It should be noted that because of the interference from VA-ECMO drainage, the measured SvO_2_ may be unreliable.

PCWP, as a surrogate marker for left ventricular end-diastolic pressure, is an important parameter for assessing left ventricular filling pressure and the effectiveness of unloading. Recent studies have proposed that the ratio of left ventricular ejection time corrected (LVETc) to PCWP, as a combined indicator integrating systolic function and filling status, can predict 30-day survival after weaning with a sensitivity of 88% and a specificity of 69% [26]. However, these results need to be validated in multicenter studies.

### 3.2 Right Ventricular-dominant Cardiogenic Shock

RV-dominant cardiogenic shock can be classified into primary and secondary types. Primary right ventricular failure is commonly seen in right ventricular infarction, postoperative complications, and similar conditions, whereas secondary right heart failure is mainly caused by increased pulmonary circulation pressure leading to right ventricular overload, such as in cases of acute massive pulmonary embolism [28]. According to data from the SHOCK trial (NCT00000552), approximately 38% of patients with acute myocardial infarction-related cardiogenic shock (AMI-CS) present with right ventricular-dominant heart failure [29]. In patients with non-ischemic cardiogenic shock, this proportion may be even higher [30]. VA-ECMO does not directly support right ventricular function, but instead indirectly supports the right heart by unloading the right ventricle and improving the coronary oxygen supply. At the same time, the increase in non-physiological blood flow may lead to elevated left ventricular afterload, which is transmitted through the pulmonary vascular system and can cause pulmonary edema, further increasing the right ventricular afterload and worsening right heart function. In this scenario, clinicians need to balance the increased ECMO flow, the resulting increase in afterload, and the potential negative impact on right heart function to ensure an appropriate treatment strategy.

When weaning off VA-ECMO to support right heart function, it is essential to reassess the objectives set for VA-ECMO. For example, in cases of massive pulmonary embolism causing acute right heart failure, VA-ECMO mainly serves as a bridge therapy; after thrombectomy surgery is completed, the right heart function may recover. In contrast, for patients whose right heart function is not expected to recover, VA-ECMO may be used as a transitional measure, serving as a bridge to a ventricular assist device (VAD) or heart transplantation.

The typical hemodynamic characteristics of right ventricular-dominant heart failure include a central venous pressure (CVP) >14 mmHg, normal or slightly elevated PCWP, and decreased cardiac index (CI) [12]. Studies have shown that the early identification of right heart failure and effective intervention can significantly reduce in-hospital mortality [29, 31, 32]. Therefore, timely diagnosis of right heart failure is crucial for improving prognosis.

The right ventricle is highly sensitive to changes in pulmonary vascular afterload, and its working capacity is approximately one-sixth of that of the left ventricle. When pulmonary artery pressure (PAP) increases (as in the case of pulmonary hypertension or pulmonary embolism), the right ventricle is prone to dilation and can easily lead to acute decompensation [33]. VA-ECMO provides indirect support to the right ventricle by unloading it through right heart drainage, thereby reducing the right ventricular wall tension and improving the delivery of oxygenated blood to the coronary circulation. However, during the weaning process, the venous return shunt effect of VA-ECMO may mask the true filling status of the right ventricle, making the assessment of right ventricular function more complex and potentially increasing the risk of right heart failure after withdrawal [18]. For patients with persistent right heart failure, transitioning to venopulmonary artery ECMO (V-PA ECMO) or a right ventricular assist device may facilitate targeted right ventricular unloading while preserving systemic oxygenation.

#### Assessment of Weaning Criteria Dominated by the Right Ventricle

At present, there is no unified standard for evaluating right ventricular function recovery or weaning timing. Under femoral artery VA-ECMO support, retrograde blood flow reconstruction may affect the interpretation of ultrasound indicators related to preload and afterload (such as tricuspid annular plane systolic excursion (TAPSE)), especially in the presence of valvular regurgitation, making ultrasound assessment even more challenging. Traditional two-dimensional ultrasound parameters, such as TAPSE, right ventricular fractional area change (RVFAC), and S’ wave, are all influenced by load dependency and may be subject to bias owing to differences in probe angle and operator technique [34, 35, 36].

In comparison, three-dimensional ultrasound (3D ultrasound) can more accurately measure right ventricular ejection fraction (RVEF), and studies have shown that an RVEF >24.6% significantly increases the likelihood of successful weaning and is closely related to 30-day survival rates [37]. However, the application of 3D ultrasound is still challenged by limitations in the imaging window and the relatively long time required for data acquisition [38]. In recent years, right ventricular-pulmonary arterial coupling (RV-PAC) has attracted attention as an important indicator of right heart function [39, 40]. The ratio of TAPSE to pulmonary artery systolic pressure (PASP) is a commonly used predictive parameter. Systematic reviews indicate that the cutoff value range for the TAPSE/PASP ratio is 0.27–0.58 mm/mmHg, with 0.36 being the most commonly used clinical standard for assessing right heart function [39]. However, the applicability of the TAPSE/PASP ratio in VA-ECMO support requires further validation. In addition, speckle-tracking echocardiography (STE) is increasingly used for the detection of right ventricular dysfunction [41, 42, 43]. A single-center prospective study involving 92 patients demonstrated that right ventricular free wall longitudinal strain (RVFWLS) >–12% was associated with higher weaning success rates [43]. The key echocardiographic and hemodynamic parameters used for ventricular function assessment during VA-ECMO weaning are summarized in Table 1 (Ref. [3, 23, 26, 37, 43, 44, 45, 46]).

**Table 1.** **Echocardiographic and hemodynamic indicators for assessing ventricular function during VA-ECMO weaning**.

	Methods	Parameters	Advantages	Limitations	References
LV	Echocardiographic	LVEF ≥20–25%	Direct marker of systolic function.	Load-dependent	Aissaoui *et al*. [3]
		LVOT VTI ≥10 cm	Direct marker of systolic function.	Load-dependent.	
		TDSa ≥6 cm/s	Load-independent.	1. Angle-dependent for valid measurement.	
				2. Interobserver variability.	
		t-IVT <14.4 seconds/minute	1. Load-independent.	1. Requires manual calculation from the filling time and ejection time.	Tavazzi *et al*. [23]
			2. Heart rate-standardized index of electromechanical efficiency.	2. Sensitive to pulse wave Doppler signal quality.	
		E/e’ <15.5	Non-invasive surrogate of left atrial pressure.	1. Limited by atrial fibrillation, valve disease, and load variability.	
				2. Tissue doppler imaging e′ is often unmeasurable.	
		MAPSE >8.9 mm	1. Bedside reproducible measure of longitudinal function.	Influenced by RV/LV interaction and may be affected in patients with acute right ventricular failure and dilatation.	
			2. Angle-independent.		
	Hemodynamic	PAWP <18 mmHg	Indirectly reflects left atrial pressure and LVEDP, useful for assessing left ventricular preload.	1. Invasive.	Aziz *et al*. [44]
				2. May not accurately reflect LVEDP in conditions such as mitral stenosis, pulmonary veno-occlusive disease, or pulmonary hypertension.	
		LVETc ∕ PAWP ≥15.9	1. A hemodynamic parameter that is based on both cardiac output and pulmonary congestion.	1. Data limited.	Sawada *et al*. [26]
			2. Shows 88% sensitivity and 69% specificity for successful VA-ECMO weaning.	2. Requires PAC insertion.	
RV	Echocardiographic	3DRVEF> 24.6%	1. Direct measurement of RV volume without geometric assumptions.	1. Time-consuming data acquisition.	Huang *et al*. [37]
			2. More accurate than 2DE.	2. Limited by loading conditions.	
				3. Requires stable patient conditions.	
				4. Offline processing limits real-time use.	
		TAPSE >16 mm	1. Simple, widely used, and reproducible.	1. Angle and operator dependent.	Tavazzi *et al*. [23]
			2. Reflects RV longitudinal function.	2. Limited by tricuspid regurgitation.	
				3. Data limited.	
		RVFWLS >–12.0% (suggest preserved RV function).	1. Angle-independent.	1. Requires high-quality images.	Gambaro *et al*. [43]
			2. Low load dependence.	2. Limited in arrhythmias or mechanical ventilation.	
			3. High reproducibility.	3. Affected by software algorithms.	
			4. Sensitive for early RV dysfunction.	4. Data limited.	
		Myocardial Work Index	1. Comprehensive assessment of myocardial efficiency.	1. Requires advanced echocardiographic techniques.	MIX-ECMO study [45]
			2. Prognostic value in VA-ECMO patients.	2. Limited by image quality and software consistency.	
				3. No standardized cutoff for VA-ECMO weaning.	
		PAPi ≥1.09 combined with rPP ≥40 mmHg predicts weaning success	1. Integration of left-ventricular and right-ventricular functional assessments.	1. Data limited.	Duong *et al*. [46]
			2. Shows 94% sensitivity and 100% specificity for successful VA-ECMO weaning.	2. Requires PAC insertion.	
				3. May be confounded by residual ECMO flow and arterial compliance changes.	

Abbreviations: 2DE, two-dimensional echocardiography; 3DRVEF, three-dimensional right ventricular ejection fraction; E/e’, early mitral inflow velocity to tissue Doppler e^′^ ratio; LV, left ventricle; LVEF, left ventricular ejection fraction; LVEDP, left ventricular end-diastolic pressure; LVETc, corrected left ventricular ejection time (LVET divided by the square root of heart rate); LVOT VTI, left ventricular outflow tract velocity-time integral; MAPSE, mitral annulus plane systolic excursion; PAC, pulmonary artery catheter; PAWP, pulmonary artery wedge pressure; PAPi, pulmonary artery pulsatility index; rPP, radial artery pulse pressure; RVFWLS, right ventricular free wall longitudinal strain; TAPSE, tricuspid annular plane systolic excursion; t-IVT, total isovolumic time; TDSa, tissue Doppler lateral mitral annulus peak systolic velocity; VA-ECMO, venoarterial extracorporeal membrane oxygenation.

### 3.3 Biventricular Dominant Cardiogenic Shock

Biventricular dominant cardiogenic shock is commonly seen in severe pathological conditions, such as end-stage heart failure, fulminant myocarditis, low cardiac output following complex surgery, and structural cardiac rupture (such as ventricular septal perforation) [47]. The hemodynamic characteristics of this phenotype include a significant elevation of both CVP and PCWP and a marked decrease in CI (<2.0 L/min/m^2^), accompanied by obvious signs of organ hypoperfusion and multiple organ dysfunction syndrome (MODS) [47]. According to reports, the failure rate of weaning for such patients can be as high as 58% [29]. Therefore, we should monitor left and right heart function separately during weaning assessment, but also pay attention to synchronization issues during the dynamic recovery process.

## 4. Weaning Trial Strategies and Procedural Protocols

Weaning from VA-ECMO is a critical process that requires careful assessment of the cardiac reserve and hemodynamic stability. Several strategies have been developed to optimize this transition, including direct trial-off or flow reduction, pump-controlled retrograde trial-off (PCRTO), and arteriovenous (AV) bridging. Each method has distinct physiological mechanisms and implications for patient outcome (Table 2, Ref. [3, 48, 49, 50]).

**Table 2.** **Comparison of different weaning trials**.

Weaning Method	Description	Advantages	Limitations	Researchers Using the Method
Direct Trial-Off	Extracorporeal life support flow was decreased to 66%, 33%, and <10% of the initial output of the device for 10 min at each level, to assess the patient’s ability to maintain circulation independently.	1. Fast.	1. Limited to hemodynamically stable patients.	Aissaoui *et al*. (2011) [3]
		2. Directly assesses whether the patient can maintain hemodynamics without ECMO.	2. High risk of sudden cardiovascular collapse.	
Stepwise Flow Reduction	Gradually reduce ECMO flow, monitor hemodynamic changes, until the minimum flow rate (usually 1–1.5 L/min) is achieved.	1. Dynamic monitoring of patient response.	1. Requires a longer time, increasing complication risks.	Pappalardo *et al*. (2015) [49]
		2. Straightforward, commonly used.	2. Limited ability to assess right heart function.	
		3. Helps assess hemodynamics.		
Arterio-Venous Bridging	Use a controlled shunt to simulate a trial of weaning while ECMO is still in place, allowing assessment of patient stability.	1. Provides a “safe” transition while monitoring circulation.	1. Requires circuit manipulation.	Pandya *et al*. (2019) [48]
		2. Reversible.	2. Higher risk of thrombosis and complications.	
Pump-Controlled Retrograde Trial-Off	Gradually reduce pump revolutions to achieve retrograde flow, providing a “stress test” for assessing cardiac function.	1. Reversible and gentle.	1. Complex setup requires specialized equipment.	Lau *et al*. (2023) [50]
		2. Helps assess right heart function and myocardial recovery.	2. Not suitable for all patients (e.g., severely impaired left ventricular function).	
		3. Applicable for a wide range of patients.		

Abbreviations: ECMO, extracorporeal membrane oxygenation.

### 4.1 Direct Trial-off or Stepwise Flow Reduction

This conventional method involves gradual ECMO flow reduction (typically 1–1.5 L/min) while monitoring end-organ perfusion. Aissaoui *et al*. [3] proposed a structured stepwise reduction protocol (66% → 33% → minimal flow) with favorable 30-day survival in successfully weaned patients. Although widely used, this approach has limitations, especially in evaluating RV function, as reduced venous return may underestimate RV capacity. Hemodynamic instability during trial-off requires immediate termination.

### 4.2 Pump-controlled Retrograde Trial-off

Originally developed in neonates, PCRTO simulates post-decannulation physiology by reversing circuit flow (0.5–1.0 L/min) from the arterial to the venous cannula. This maneuver reduces LV afterload while increasing RV preload, allowing the assessment of the biventricular reserve under near-physiological conditions. The trial typically lasts 30–120 min and is guided by echocardiography; when available, PAC can provide real-time metrics such as pulmonary artery wedge pressure (PAWP) and cardiac index.

In a single-center study (n = 20), Xu *et al*. [51] reported that elevated PAWP during PCRTO predicted weaning failure, underscoring its potential utility in early risk stratification. Other parameters, including central venous pressure, lactate level, and urine output, should also be monitored to assess systemic perfusion. Circuit management is critical; zero-flow modes and air embolism risks must be avoided using standardized protocols.

PCRTO offers procedural reversibility and standardization with a low complication rate. Retrospective data from Jo *et al*. [52] suggest improved discharge survival compared to conventional flow reduction; however, its evidence base remains limited by small sample sizes and the lack of randomized trials. Further prospective studies are required to validate its role in adult VA-ECMO weaning protocols.

### 4.3 Arterio-venous Bridging

AV bridging involves temporarily connecting the arterial and venous limbs of the ECMO circuit to preserve circuit flow and to prevent blood stagnation during weaning. Hemodynamic and respiratory monitoring are essential throughout the procedure. In the event of instability manifested by elevated lactate levels, increased inotrope requirements, fluid overload, or hypercapnia, ECMO can be promptly reinstated by removing the bridge.

Compared with PCRTO, AV bridging shows similar weaning success but lower discharge survival and longer trial durations [48]. The method is reversible and technically straightforward, but manipulation of the circuit introduces thrombotic risks. Strategies, such as intermittent clamp release and heparin flushing, may mitigate these concerns, although thromboembolism remains a limitation. Therefore, close monitoring and strict anticoagulation are essential.

## 5. Cannulation Strategies and Implications for Weaning

The cannulation approach affects left ventricular afterload, hemodynamics, the reliability of echocardiographic assessments, perfusion distribution, and the risk of complications during decannulation. Understanding these differences is crucial for individualized weaning decisions. This chapter mainly discusses cannulation methods for VA-ECMO, including femoral-femoral, axillary artery, and central cannulation.

### 5.1 Femoral Artery Cannulation

In most cases, peripheral arterial cannulation is the most commonly used method because it is the least complex to perform and is suitable for bedside insertion in emergency situations [53, 54, 55]. However, this method of catheterization requires moving against the direction of arterial blood flow to the aortic root, which greatly increases the afterload on the left ventricle. As a result, the aortic valve may fail to open spontaneously, leading to left ventricular dilation and worsening pulmonary congestion, especially in patients with poor baseline myocardial contractility [13]. The incidence of differential hypoxemia (Harlequin syndrome) is higher in such cases, especially when there is pre-existing pulmonary dysfunction. Sorokin and colleagues [54] reported a limb ischemia rate as high as 10%, and in the absence of a distal perfusion strategy, the risk of amputation approaches 5%.

For the purpose of weaning, VA-ECMO support may obscure the true extent of myocardial recovery due to increased afterload, so it is necessary to use adjunctive left ventricular unloading strategies (such as IABP or percutaneous axial flow pump (e.g., Impella)) to enable meaningful decannulation assessment.

### 5.2 Subclavian Artery/Axillary Artery Catheterization

Subclavian or axillary artery cannulation can serve as a form of peripheral access. This approach may be used for patients with peripheral vascular disease or for whom femoral artery access is extremely difficult, in order to prevent vascular cannulation complications at the femoral artery site, including lower limb ischemia, bleeding, vascular perforation or rupture, and inadequate cannula size [25]. Subclavian artery cannulation is typically performed surgically and provides antegrade perfusion that more closely resembles physiological aortic flow. Compared to femoral artery cannulation, subclavian or axillary artery cannulation results in lower left ventricular afterload, promotes spontaneous aortic valve opening, and improves left ventricular ejection. Additionally, subclavian or axillary artery cannulation is associated with a lower rate of left heart unloading compared to femoral artery cannulation [53, 56]. However, in a multicenter retrospective analysis of the Post-Cardiotomy Extracorporeal Life Support (PELS) registry (n = 1897), subclavian/axillary arterial cannulation was independently associated with a higher incidence of major neurologic complications—composite of ischemic stroke, cerebral hemorrhage, and brain oedema—compared with femoral cannulation (19.6 % vs 11.9 %; adjusted odds ratio (OR) 1.53, 95 % CI 1.02–2.31, *p* = 0.041), despite adjustment for peripheral artery disease, prior stroke, hypertension, and other confounders [53].

Importantly, antegrade blood flow allows for a more accurate echocardiographic interpretation of left ventricular recovery, including valve movement and LVOT VTI. However, the subclavian approach requires surgical expertise and carries the risk of upper limb hyperperfusion or compartment syndrome. Sorokin and colleagues [54] emphasized that ischemic complications of the upper limb occur in up to 20% of cases, necessitating meticulous surgical technique and vigilant monitoring. For patients who are expected to receive prolonged support and have potential for myocardial recovery, subclavian cannulation is the preferred choice due to its physiological advantages and benefits related to weaning.

### 5.3 Central Catheterization

Central cannulation through direct aortic and right atrial access is most commonly used in post-cardiotomy settings or when peripheral access is inadequate [54]. This configuration provides the most effective anatomical and hemodynamic support: antegrade perfusion minimizes left ventricular afterload, promotes aortic valve opening, and maximizes the fidelity of echocardiographic recovery assessments.

Immediate decompression of both ventricles via central inflow and outflow allows reliable monitoring of native cardiac function during flow reductions or trials. In addition, transesophageal echocardiography (TEE) is easily performed during open chest or perioperative periods, further enhancing assessment accuracy. However, central cannulation requires surgical exposure, increases bleeding risk, and is not suitable for percutaneous or emergency implementation. For postoperative patients in a controlled surgical setting, central cannulation is the ideal choice for early recovery assessment and precise decannulation planning.

In summary, the cannulation strategy chosen at the initiation of VA-ECMO has a profound impact on weaning. Although the femoral-femoral approach is expedient, it may mask or worsen left ventricular dysfunction; the subclavian route improves physiological blood flow and imaging accuracy; central cannulation offers the greatest decompression and monitoring capability, but is limited to surgical candidates. Weaning protocols must take these differences into account in order to accurately assess cardiac recovery and avoid premature decannulation.

## 6. Pharmacologic and Volume Management in VA-ECMO Weaning.

### 6.1 Vasoactive Agents and Levosimendan

Vasoactive medications are essential for circulatory support during VA-ECMO; however, elevated vasoactive-inotropic scores are associated with poor outcomes, including reduced weaning success and higher mortality [57, 58]. Levosimendan, a calcium sensitizer, enhances myocardial contractility without increasing oxygen demand and has vasodilatory and anti-ischemic properties. Despite its theoretical benefits, recent evidence questions its clinical utility [59, 60, 61, 62, 63]. A propensity-matched analysis of patients with refractory cardiogenic shock showed no significant difference in weaning success (45% vs 34%) or 6-month survival between levosimendan and control groups, despite improved LV function metrics [64]. These findings, adjusted for immortal time bias, contrast with earlier observational data and highlight the need for high-quality randomized trials, such as the ongoing LEVOECMO study (NCT04728932).

### 6.2 Volume Management

Volume overload is common in VA-ECMO patients and independently predicts reduced weaning success, organ recovery, and long-term survival [65, 66, 67]. The 2021 ELSO guidelines advocate the gradual achievement of a negative fluid balance during weaning [25]. Retrospective data suggest that a cumulative fluid balance of >38.8 mL/kg within 24 h, or persistent overload by day 3, significantly increased 90-day mortality (OR 4.02, *p* = 0.006) [68].

Assessment of volume status is challenging because of capillary leak and hemodilution, necessitating a multimodal approach. Tools included echocardiography (IVC diameter, LV function), pulse pressure, and sublingual microcirculatory imaging. However, ECMO non-physiological flow limits the predictive accuracy of these measures [69]. Diuretics remain the first-line treatment for fluid removal, whereas renal replacement therapy (RRT) is used in resistant cases or for fine control [25].

### 6.3 Inhaled Nitric Oxide (iNO)

Inhaled nitric oxide selectively reduces pulmonary vascular resistance, thereby lowering the RV afterload and improving RV function without systemic hypotension. Its application in VA-ECMO, particularly in patients with RV dysfunction or pulmonary hypertension, may enhance biventricular performance and support safe weaning [70, 71, 72, 73]. Evidence from myocardial infarction and cardiac arrest models suggests that iNO may also confer neuroprotection and aid in hemodynamic stabilization [74]. However, its effect on survival remains inconclusive and warrants further investigation.

## 7. Stepwise Evaluation of Weaning Readiness in VA-ECMO Patients

Successful weaning from VA-ECMO depends on the recovery of myocardial function, end-organ perfusion, and hemodynamic stability, all of which can be assessed using a combination of biomarkers, microcirculatory monitoring, and echocardiographic evaluation.

Hemodynamic indicators such as PP serve as surrogate markers of cardiac contractility. Recent studies suggest that early improvement in PP correlates with better outcomes in patients with cardiogenic shock supported by VA-ECMO [75, 76, 77]. Particularly in acute myocardial infarction-related shock, the PP adjusted by vasoactive-inotropic score (PP/$\sqrt{V\,I\,S}$) within 12 hours after ECMO initiation has demonstrated promising predictive value for weaning success (sensitivity 67.1%, specificity 86.1%) [76].

Lactate levels and clearance rates are commonly used to reflect global tissue perfusion [78, 79, 80]. A multicenter retrospective study involving 685 VA-ECMO patients found that higher lactate clearance within 24 h was significantly associated with successful weaning (OR 0.21, 95% CI 0.10–0.44, *p* < 0.001) [80]. Notably, lactate levels may be influenced by various non-cardiac factors, such as impaired liver function, highlighting the need for a multidimensional assessment approach.

Recent evidence has highlighted the prognostic value of microcirculatory parameters such as perfused small vessel density (PSVD), perfused vessel density (PVD), and the proportion of perfused vessels (PPV), which appear to outperform traditional markers such as lactate in predicting weaning outcomes [81, 82]. The preservation of microcirculatory function during ECMO flow reduction is strongly associated with successful weaning and lower short-term mortality. Although guidelines for sublingual microcirculation assessment were introduced by the European Society of Intensive Care Medicine (ESICM) in 2018 [83], their clinical adoption remains limited. More prospective studies are needed to validate its routine use in patients on VA-ECMO.

**Fig. 1.**
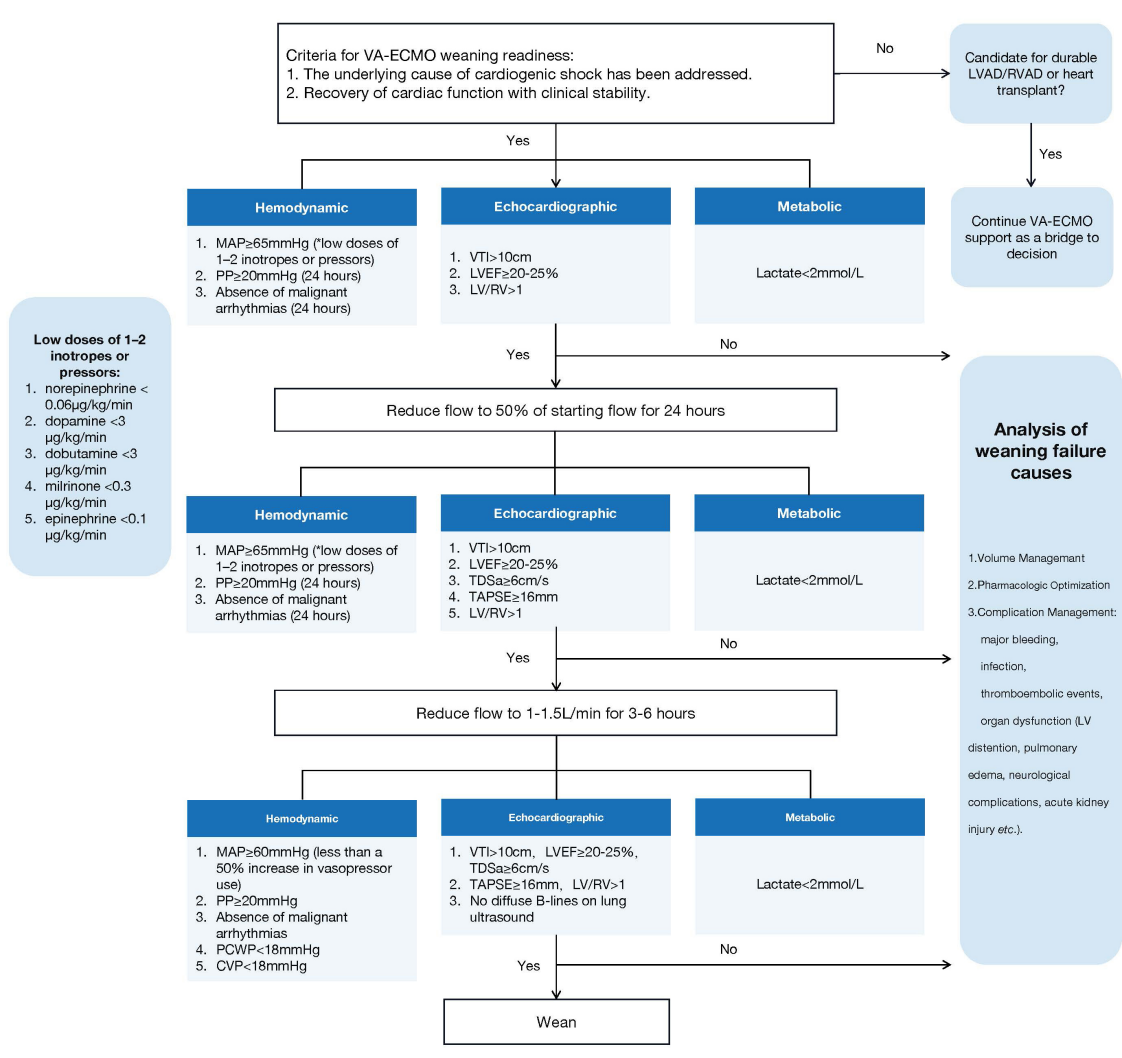
**A structured and stepwise weaning protocol**. Abbreviations: CVP, central venous pressure; LV, left ventricle; LVAD, left ventricular assist device; LVEF, left ventricular ejection fraction; MAP, mean arterial pressure; PCWP, pulmonary capillary wedge pressure; PP, pulse pressure; RV, right ventricle; RVAD, right ventricular assist device; TAPSE, tricuspid annular plane systolic excursion; TDSa, tissue Doppler systolic velocity at the lateral mitral annulus; VA-ECMO, venoarterial extracorporeal membrane oxygenation; VTI, left ventricular outflow tract velocity time integral.

At our center, we adopted a structured and stepwise weaning protocol that integrates hemodynamic, echocardiographic, and metabolic parameters to assess readiness and guide weaning from VA-ECMO support (Fig. 1).

Step 1: Weaning readiness assessment.

When the underlying cause of cardiogenic shock has been addressed and myocardial function has recovered with clinical stability, weaning from VA-ECMO is initiated. If these conditions are not met, reassessment is required, including evaluation of the volume status, pharmacologic optimization, and consideration of left ventricular unloading or the presence of pericardial effusion. If cardiac recovery is deemed unlikely, patients should be evaluated for durable mechanical support, such as a left ventricular assist device, right ventricular assist device implantation, or heart transplantation.

Step 2: Initial assessment under the starting flow.

Under starting VA-ECMO flow, we assess:

• Hemodynamics: MAP ≥65 mmHg maintained with low-dose vasopressors/inotropes continued for 24 hours, PP ≥20 mmHg continued for 24 hours, and absence of malignant arrhythmias for over 24 hours.

• Echocardiography: LVOT VTI >10 cm, LVEF ≥20–25%, and LV RV diameter ratio >1.

• Metabolic status: Lactate <2 mmol/L.

Step 3: Assessment under half flow.

If all criteria were met, flow was reduced to 50% of the initial setting for 24 h. Under VA-ECMO support with a flow of half flow and the use of 1–2 vasoactive agents, reassessment of echocardiographic findings, hemodynamic parameters, and metabolic indicators is required. Additional echocardiographic parameters such as TDSa ≥6 cm/s and TAPSE ≥16 mm were included.

If stable, the flow is further reduced to 1–1.5 L/min for 3–6 h.

Step 4: Final pre-weaning evaluation.

Under minimal flow:

• Hemodynamic targets: MAP ≥60 mmHg with less than <50% increase in vasopressor use, PP ≥20 mmHg, PCWP, and CVP <18 mmHg.

• Echocardiography: Maintenance of prior criteria with no diffuse B-lines on lung ultrasound to rule out pulmonary congestion, as persistent pulmonary dysfunction may lead to complications such as Harlequin syndrome or severe hypoxemia.

• Metabolic: Lactate remains <2 mmol/L.

Patients fulfilling all criteria are considered eligible for safe decannulation.

This stepwise approach allows for gradual tapering of mechanical support while ensuring real-time assessment of native cardiac function. By combining multiple physiological domains, it mitigates the limitations of single-parameter evaluation (e.g., load-dependent echo measures) and highlights potential reasons for weaning failure (e.g., complications like bleeding or thrombosis). The protocol emphasizes a cautious but progressive flow reduction strategy with close monitoring. It is generalizability requires prospective validation across different shock phenotypes and institutions.

## 8. Management of Weaning Failure and Associated Complications

Weaning failure from VA-ECMO is commonly linked to complications or insufficient recovery of cardiac and organ function [6, 84]. Management should therefore emphasize complication control and timely adjustment of mechanical support to enhance the likelihood of successful liberation.

### 8.1 Complication Management During Weaning

Complications associated with VA-ECMO represent major determinants of both weaning success and long-term outcomes [6, 85]. A meta-analysis of 20 studies involving 1866 patients demonstrated high complication rates, including acute kidney injury (55.6%), major bleeding (40.8%), severe infection (30.4%), limb ischemia (16.9%), and neurological events (13.3%) [85]. In addition, a systematic review by Makhoul *et al*. [6] highlighted that multiple organ failure is the predominant cause of mortality following weaning, whereas bleeding remains the most frequent and fatal complication during ECMO support. These findings underscore the critical importance of early detection and continuous monitoring of complications to improve patient prognosis.

Significant bleeding events during VA-ECMO support frequently involve internal organs, intracranial regions, and cannulation sites. Their occurrence is strongly associated with systemic anticoagulation, dilution and consumption of coagulation factors, hepatic and renal dysfunction, and underlying disseminated intravascular coagulation [25]. Beyond routine monitoring of hemoglobin levels and coagulation profiles, clinicians should be attentive to hemodynamic instability and increasing requirements for vasoactive drugs, as these may suggest the presence of unrecognized or significant bleeding.

During phases of reduced pump flow and weaning trials, meticulous adjustment of anticoagulation—typically guided by activated clotting time—is essential to minimize thrombus formation within the extracorporeal circuit and cardiac chambers, thereby reducing the risk of limb ischemia and cerebral embolism [25]. Neurological complications may arise from differential hypoxemia, particularly in patients with peripheral femoral cannulation when myocardial and pulmonary recovery are asynchronous [25, 86]. Limb ischemia, characterized by absent arterial pulses, pallor, or reduced skin temperature, often results from large-bore cannulas or intraluminal thrombosis and demands prompt recognition and intervention to prevent irreversible injury. Monitoring right radial arterial blood gases and, when available, near-infrared spectroscopy of cerebral oxygenation and lower limbs can facilitate timely detection [25, 86]. Infection is another frequent complication, exacerbated by indwelling catheters and extracorporeal circuits. Notably, fever may be masked by the use of heat exchangers, emphasizing the importance of routine surveillance of inflammatory markers for timely diagnosis. Acute kidney injury, occurring in more than half of VA-ECMO patients, may result not only from systemic hypoperfusion but also from hemolysis and the non-pulsatile nature of extracorporeal flow. Therefore, close monitoring of urine output, urine color, and plasma-free hemoglobin levels is warranted.

Collectively, these considerations underscore that rigorous surveillance, early recognition, and targeted management of complications are integral to improving the likelihood of successful liberation from VA-ECMO.

### 8.2 Adjustment of Mechanical Support

While vigilant management of complications is essential for ensuring patient safety during VA-ECMO weaning, failure to achieve sustained hemodynamic stability or incomplete recovery of cardiac function often necessitates adjustments in mechanical support. In such scenarios, adjunctive unloading devices or bridging strategies provide important therapeutic options to optimize outcomes [87].

#### 8.2.1 Left Ventricular Unloading

As discussed earlier, unloading strategies target left ventricular afterload reduction, myocardial wall stress, coronary perfusion, and pulmonary congestion. A combined approach incorporating sequential weaning and adjunctive unloading devices, such as IABP or Impella in conjunction with VA-ECMO, may offer transitional support during recovery from biventricular dysfunction [88, 89]. IABP remains the most widely available unloading device, providing modest afterload reduction and augmentation of diastolic coronary flow [13]. Some studies suggest that a PCWP exceeding 15–18 mmHg prior to VA-ECMO cannulation may predict a favorable response to IABP unloading [87, 90]. When VA-ECMO is combined with IABP, weaning typically prioritizes the removal of VA-ECMO first, owing to its potential to increase left ventricular afterload and its relatively high complication risk [13]. However, the specific indications, optimal timing, and clinical benefits of IABP remain to be clarified and require further validation in prospective studies [87, 89, 91].

The combination of Impella with VA-ECMO—commonly referred to as the ECmella strategy—has gained increasing attention. This approach offers more robust ventricular unloading compared with IABP, actively reducing afterload and oxygen demand, while enhancing coronary perfusion and alleviating pulmonary congestion [13, 92]. The DanGer Shock trial (NCT01633502) recently provided the first randomized evidence supporting the use of microaxial flow pumps in patients with acute myocardial infarction complicated by cardiogenic shock, demonstrating improved survival at 180 days. However, an increased risk of bleeding was also observed, underscoring the need for careful patient selection and monitoring [93].

#### 8.2.2 Bridge to Durable Mechanical Support or Transplant

VA-ECMO provides a crucial therapeutic window in patients with end-stage heart failure, enabling clinicians to assess the potential for myocardial recovery and to formulate subsequent treatment strategies. When recovery is unlikely, bridging to durable left ventricular assist devices (dLVADs) or heart transplantation should be considered.

Policy reforms have significantly influenced bridging strategies. For example, the 2018 revision of the United Network for Organ Sharing (UNOS) heart allocation system granted VA-ECMO the highest emergency priority, thereby increasing access to transplantation for these critically ill patients [94]. Despite this advantage, concerns remain. VA-ECMO has been associated with greater transfusion requirements and an elevated risk of perioperative bleeding, which may negatively impact post-transplant outcomes [95].

When transplantation is not immediately feasible or donor organs are limited, bridging VA-ECMO to dLVAD implantation represents a viable alternative. Analysis of INTERMACS and UNOS registry data demonstrated comparable long-term survival between patients bridged with VA-ECMO to dLVAD and those bridged directly to transplantation (5-year survival 43.5% vs 38.2%, *p* = 0.581) [96]. Moreover, in selected patients with pulmonary hypertension and right heart failure, studies indicate that outcomes following combined heart–lung transplantation are similar to those achieved with isolated bilateral lung transplantation [97], suggesting that right ventricular function may recover once elevated afterload is relieved.

Taken together, these findings highlight the importance of clearly defining the intended goal of VA-ECMO initiation—whether as a bridge to recovery or to transplantation. A tailored evaluation of cardiopulmonary function is therefore essential to guide subsequent supportive strategies and to optimize patient outcomes following VA-ECMO weaning.

## 9. Conclusion

This review underscores the multifactorial determinants of successful weaning from VA-ECMO in patients with refractory cardiogenic shock. Weaning should be regarded not as a discrete event but as a structured and iterative process that reflects the dynamic evolution of each patient’s clinical course. Comprehensive evaluation of myocardial recovery—including biventricular function, hemodynamic stability, and metabolic status—is fundamental to guide decision-making. Tailoring strategies to ventricular phenotypes (left-, right-, or biventricular-dominant failure), combined with advanced monitoring, pharmacologic optimization, and volume management, facilitates a more individualized approach. Equally important is the timely recognition and management of complications, as well as consideration of transitions to adjunctive support such as IABP or Impella, or to durable options including dLVAD or transplantation. Standardized protocols and validated predictive tools remain lacking, underscoring the need for multicenter studies to establish phenotype-specific weaning criteria. Advancing these efforts will be crucial to improving outcomes in this high-risk population.
